# Relationship between clinical signs and postmortem test status in cattle experimentally infected with the bovine spongiform encephalopathy agent

**DOI:** 10.1186/1746-6148-6-53

**Published:** 2010-12-09

**Authors:** Timm Konold, A Robin Sayers, Amanda Sach, Gemma E Bone, Steven van Winden, Gerald AH Wells, Marion M Simmons, Michael J Stack, Angus Wear, Steve AC Hawkins

**Affiliations:** 1 Veterinary Laboratories Agency Weybridge, Host Pathology and Susceptibility Department, New Haw, Addlestone, KT15 3NB, UK; 2 Veterinary Laboratories Agency Weybridge, Centre for Epidemiology and Risk Analysis, New Haw, Addlestone, KT15 3NB, UK; 3 ADAS Drayton, Alcester Road, Stratford upon Avon, CV37 9RQ, UK; 4 Royal Veterinary College, Department of Veterinary Clinical Sciences, North Mymms, Hatfield, AL9 7TA, UK; 5 Veterinary Laboratories Agency Newcastle, Laboratory Services, Longbenton, Newcastle-upon-Tyne, NE12 9SE, UK

## Abstract

**Background:**

Various clinical protocols have been developed to aid in the clinical diagnosis of classical bovine spongiform encephalopathy (BSE), which is confirmed by postmortem examinations based on vacuolation and accumulation of disease-associated prion protein (PrP^d^) in the brain. The present study investigated the occurrence and progression of sixty selected clinical signs and behaviour combinations in 513 experimentally exposed cattle subsequently categorised postmortem as confirmed or unconfirmed BSE cases. Appropriate undosed or saline inoculated controls were examined similarly and the data analysed to explore the possible occurrence of BSE-specific clinical expression in animals unconfirmed by postmortem examinations.

**Results:**

Based on the display of selected behavioural, sensory and locomotor changes, 20 (67%) orally dosed and 17 (77%) intracerebrally inoculated pathologically confirmed BSE cases and 21 (13%) orally dosed and 18 (6%) intracerebrally inoculated but unconfirmed cases were considered clinical BSE suspects. None of 103 controls showed significant signs and were all negative on diagnostic postmortem examinations. Signs indicative of BSE suspects, particularly over-reactivity and ataxia, were more frequently displayed in confirmed cases with vacuolar changes in the brain. The display of several BSE-associated signs over time, including repeated startle responses and nervousness, was significantly more frequent in confirmed BSE cases compared to controls, but these two signs were also significantly more frequent in orally dosed cattle unconfirmed by postmortem examinations.

**Conclusions:**

The findings confirm that in experimentally infected cattle clinical abnormalities indicative of BSE are accompanied by vacuolar changes and PrP^d ^accumulation in the brainstem. The presence of more frequently expressed signs in cases with vacuolar changes is consistent with this pathology representing a more advanced stage of disease. That BSE-like signs or sign combinations occur in inoculated animals that were not confirmed as BSE cases by postmortem examinations requires further study to investigate the potential causal relationship with prion disease.

## Background

Classical BSE is a prion disease of domesticated cattle presenting as a slowly progressive neurological disorder [1]. Signs associated with BSE, which comprise abnormalities of behaviour, sensation and locomotion, have been assessed as part of the passive surveillance in the UK to define the clinical phenotype of BSE [2,3]. Based on more detailed examinations and observations of BSE field cases, specific clinical protocols have been developed to aid in the clinical diagnosis of BSE field suspects [4-6] and to monitor clinical onset and progression in cattle experimentally challenged with BSE [7].

The confirmatory diagnosis of BSE was initially reliant on detection of vacuolar changes or scrapie-associated fibrils in the brain [1]. It was shown that in clinically suspect cases lesions occurred consistently in the brainstem and that examination of the medulla oblongata provided the greatest sensitivity of the histopathological diagnosis [8]. Correlation of the histopathological changes, visualisation of fibrils and the detection of the pathognomonic abnormal isoform of the prion protein (PrP^Sc^) by Western blotting, dot and histo-blotting confirmed the suitability of sampling the medulla for diagnosis [9,10]. In the past decade refinements in the regulatory diagnosis have fully implemented disease-specific immunological methods including IHC, for detection of the disease-associated prion protein, PrP^d^, in tissue sections without the use of protease enzymes and WB and ELISA tests, which detect proteinase K resistant prion protein, PrP^res ^[11,12].

The currently used postmortem tests can diagnose a TSE in the preclinical stage, before the animal displays evident signs of a neurological dysfunction, and this has been shown in experimentally induced BSE, where the term "definite signs" has been used to denote clearly progressive neurological disease [13]. Earlier changes, mostly behavioural, may be present [14] but due to their unspecific nature may overlap with the range of expressions of normal animal behaviour.

In contrast to field cases of BSE where the time of exposure to the causative agent via contaminated MBM is not known and BSE-like signs can be due to various other diseases [15], experimental challenges with the BSE agent offer the opportunity for regular clinical monitoring of animals under controlled conditions, enabling detailed documentation of clinical signs and subsequent correlation with the results of confirmatory diagnostic tests.

This study investigates whether clinical signs associated with BSE (based on certain selected test responses or their combinations) are specific for pathologically confirmed cases of experimental BSE in cattle and can be displayed in the absence of detectable vacuolation, PrP^d ^or PrP^res ^in the brainstem by currently applied diagnostic postmortem examinations.

## Methods

All procedures were carried out in accordance with the Animal (Scientific Procedures) Act 1986, under licence from the UK Government Home Office.

### Animal data sources

The data used in this study were derived from the monitoring of cattle from two previously reported experiments which examined the transmissibility of BSE [13,16]. In study 1 [13], 200 Friesian cattle were dosed orally; 90 castrated males and ten female cattle with 100 g and 90 castrated male and ten female cattle with 1 g of titred BSE-affected brainstem homogenate. One hundred Friesian undosed cattle (90 castrated male and ten female cattle) served as controls. All cattle were purchased from UK farms with no history of BSE at the time of sourcing. (Subsequently, three BSE cases from two source farms were detected as part of the BSE surveillance; one of these was sold off to another farm. These cases were born between 1992 and 1994, whereas 12 cattle from these farms that were used for the project were born in 1998). Each group was housed separately. Heifers were artificially inseminated to produce calves and enable sequential milk collections. Steers were subject to regular sampling of blood and cerebrospinal fluid for archiving purposes and were culled by random allocation to a sequential kill protocol: six challenged and three age-matched control cattle were killed at three-monthly intervals after dosing, increasing to six-monthly intervals after dosing subsequent to the first year in the case of the 1 g dose group. Female cattle were kept until development of unequivocal signs of a neurological disease consistent with BSE or signs of any other untreatable or welfare compromising disease; surviving dosed cows were culled at 88 mpi together with age-matched control cows.

Data from 291 cattle was used for the analysis; excluded animals were predominantly controls culled early in the course of the experiment. The diagnostic outcome of cattle from this study has been reported previously [13] and further information on individual animals published elsewhere [17].

In study 2 [16], 63 groups of five Friesian or Friesian crossbred castrated male calves from UK farms with no history of BSE at the time of sourcing were inoculated intracerebrally. (Seven BSE cases from four source farms were detected subsequently as part of the BSE surveillance; five of these were sold off and one was purchased. These cases were born between 1987 and 1994, whereas the earliest birth year of 65 cattle that were sourced from these farms for the project was 1996). Each of 62 groups received a different pool of tissues or body fluids (1 ml of a 10% homogenate in saline solution) obtained from calves dosed orally with 100 g of titred BSE-affected brainstem homogenate and culled at various time points during the incubation period [16,18]. The remaining group received a pooled inoculum of nictitating membrane from naturally infected BSE cases [19]. Two groups of Friesian or Friesian crossbred male castrated cattle inoculated with saline solution served as controls. The groups were kept separately. Cattle were retained for seven years post inoculation or culled when they developed BSE signs or other untreatable or welfare compromising disease. Data from all cattle of study 2 were analysed. The diagnostic status of individual cattle at the termination of this study ([20]; SAC Hawkins and GAH Wells, unpublished data), differed from that in the published interim report of the experiment [16] in only the outcome of an additional tissue inoculation with 1/5 cattle positive that were inoculated with a pool of nictitating membrane from natural cases of BSE.

### Clinical monitoring

Clinical signs are defined as behavioural or other signs that are observed irrespective of an underlying pathological condition. Some signs may - to an extent - also be displayed by healthy animals, such as nose licking or over-reactivity to external stimuli. The clinical assessments in this study comprised neurological examinations and behavioural observations.

#### Neurological examinations

Neurological examinations were carried out every three months from 12 mpi according to a standard protocol [7]. Pre-cull examinations were generally carried out in the week prior to euthanasia of the animals. Tests of over-reactivity were used to assess responses to external stimuli and included the stick test, the flash test, clipboard test and bang test or hand clap as previously described [5].

#### Behavioural observations

Regular, usually weekly, passive observations of cattle in their pens were carried out by trained scientific staff or veterinarians. The observation time was dependent on the group size, calculated as 3 minutes multiplied by the number of animals in the group, but was usually 15 minutes, even for groups of less than five animals. A standard form was used to record behaviours associated with BSE [7].

As far as was possible all assessments were made without the observer having prior knowledge of the inoculation status of the cattle.

### Animal husbandry

Cattle were housed in medium security accommodation at two facilities: VLA Weybridge and ADAS Drayton. The latter housed all cattle in study 1 and 47 groups of cattle in study 2. These cattle were usually transported to Weybridge prior to euthanasia and a pre-cull examination was conducted at least one day after transport so that they could acclimatise to the new environment. All cattle were given straw or hay *ad libitum *and all received a daily concentrate ration free from MBM. The amount of hay or straw and concentrate ration varied according to growth of the cattle and their decreasing energy demand with increasing age to avoid excessive obesity, particularly in steers. Similarly, individual cattle within a group received food supplement if their weight or body condition score decreased considerably compared to the rest of their group. Cows, which were used for milk production, were fed according to their energy requirements during lactation and dry period. This resulted in fluctuation of the weight and body condition of cattle and made assessment of these signs too unreliable to be used for the analysis.

### Postmortem diagnosis

The tests used for BSE diagnosis have been described elsewhere [13,21] and included those used for the statutory confirmation of BSE in the UK surveillance programme. Sections of the medulla at the obex were stained with haematoxylin and eosin for HP and immunolabelled with antibody R145 for detection of PrP^d ^by IHC. WB used the "VLA Hybrid" technique [22], and an ELISA method (BioRad TeSeE [13]) was used on fresh samples of the caudal medulla adjacent to the obex to detect PrP^res^.

Further sections of brainstem from all cattle in study 1, representing rostral medulla oblongata and mesencephalon, immunolabelled with antibody R145, were examined in a previous study [23]. In addition to further brainstem sections, cerebellum, thalamus, corpus striatum and cerebral cortex were similarly examined from all cattle in study 2 as described previously for selected cases [21].

### Analysis

#### Clinical signs

The clinical signs that were evaluated and their corresponding definitions are displayed in the Additional file 1: Assessed signs. These included 27 signs assessed by clinical examinations and 33 signs assessed on behavioural observations. Behavioural observations were passive and were conducted more frequently than clinical examinations, which included active stimulation of the animals. The two approaches were compared separately, even if some of the assessed signs were identical. The selection of clinical signs was based on published descriptions of signs in natural cases of BSE in cattle [3,24,25]. Not all signs could be assessed in all cattle for various reasons. For example, recognition of the significance of certain clinical tests resulted in their subsequent application during the study (e.g. tests of over-reactivity). Also signs like "nervous entering the milking parlour" were only assessed in study 1; the milking parlour being available only in that study and used simply to evaluate the behaviour of steers in an unfamiliar environment.

All signs were considered to be independent although some combinations of signs were also considered. For example, as the assessment of over-reactivity to external stimuli has been particularly useful in the clinical diagnosis of suspect BSE cases [5,24], the responses to hand clap or bang test, flash test, clipboard test, stick test were combined as tests of over-reactivity. In addition, the single abnormal response to sudden noise, flash light and the clipboard and the single occurrence of startle responses in general was assessed separately from repeated (more than twice) events.

As the reliable clinical diagnosis of BSE has been made when cattle display evident signs in at least two of the three categories: changes in behaviour, sensation and locomotion [24], selected signs in these categories, noted at the pre-cull examination and the last three observation periods prior to cull, were assessed. Criteria for the suspect diagnosis of BSE were the combined display of at least one of the signs in two of the following three categories:

• Behavioural changes: nervousness or apprehension in the corridor (during the clinical examination) or apprehension (during the observation period).

• Sensory changes: Abnormal stick test, repeated (more than twice) over-reactivity to external stimuli (clipboard test, flash test, test to over-reactivity to sound) or spontaneous repeated startle responses (during the examination) or spontaneous startle responses, flinch on approach by hand, repeatedly in one observation period or once each in least two observation periods.

• Locomotor changes: Ataxia or hypermetria.

### Statistical analysis

Grouping of animals for analysis according to source study, experimental treatments and the total number of assessments (examinations and observations) throughout the study are given in Table 1. Groupings were according to the postmortem BSE diagnosis, with subgroupings depending on the inoculation dose (study 1) or tissue type inoculated (study 2). For comparison, animals of study 1 were grouped into undosed controls, BSE-negative challenged cattle and BSE-positive challenged cattle. In addition, as confirmed BSE cases were culled at different disease stages, cases with a positive IHC postmortem result (presence of PrP^d^) were further divided into those with evident vacuolar changes and those with no or only minimal vacuolar changes at the obex (not shown in Table 1). Animals in study 2 were grouped into control cattle (group 1), those negative on postmortem test diagnosis that were inoculated with tissues that were not expected to be infectious because of published information on tissue infectivity relative to incubation period in BSE pathogenesis (group 2), those negative on postmortem test diagnosis but with expected infectivity either because infectivity has been previously demonstrated in the donor tissue [23,26-28] or other animals in the group were positive for BSE on postmortem test diagnosis (group 3) and cattle positive on postmortem test diagnosis that were inoculated with tissues with confirmed infectivity (group 4). The grouping was made under the assumption that the clinical presentation would be independent of the inoculated tissue that produced the disease because BSE in cattle inoculated with distal ileum, tonsil or CNS appeared to be clinically similar (T Konold, unpublished observation).

**Table 1:** Grouping of animals according to source study, experimental treatments, postmortem diagnosis, the total number of assessments (examinations and observations) and the number (%) clinically diagnosed on the basis of selected criteria prior to cull

Group (N animals)	Postmortem BSE diagnosis	Total examinations	Total observations	Number (%) of cattle with a clinical diagnosis of BSE
**Study 1 (oral challenge)**				

Controls, not dosed (93)	Negative	982	8 473	0 (0)

100 g BSE brainstem (25)	Positive	318	2 338	17 (68)

1 g BSE brainstem (5)	Positive	96	923	3 (60)

100 g BSE brainstem (74)	Negative	365	2 139	11 (15)

1 g BSE brainstem (94)	Negative	1 076	9 205	10 (11)

**Study 2 (intracerebral inoculation)**				

Group 1: Control (10)	Negative	193	2 556	0 (0)

Group 2: Tissue without expected infectivity (240)	Negative	5 290	59 310	15 (6)*

Group 3: Tissue with expected infectivity (53)	Negative	1 204	12 526	3 (6)*

Group 4: Tissue with confirmed infectivity (22)	Positive	110	1 051	17 (77)

**Total**		**9 634**	**98 521**	

Study 1: cattle culled at pre-determined time points post dosing.

Study 2: cattle culled upon development of BSE or at 7 years post inoculation; see Methods for groupings.

* Clinical suspects despite negative postmortem BSE diagnoses were: in group 2 (n = 15) inoculated with pooled kidney (collected at 18 mpi), mesenteric lymph node (18 mpi), bone marrow (36 mpi), buffy coat (18 mpi), buffy coat (26 mpi; n = 2), CNS and spinal cord C2-3 & T10-11 (18 mpi; n = 2), cervical/popliteal lymph nodes (26 mpi), peripheral nerve (26 mpi), pooled liver (32 mpi), salivary gland (18 mpi), skin (18 mpi), spleen (18 mpi) and urine (18 mpi) [16], and

in group 3 (n = 3) inoculated with distal ileum (collected at 26 mpi), tonsil (6 mpi) and caudal medulla/spinal cord (26 mpi).

The study 2 groups were made up as follows:

Group 1 (control): cattle inoculated with saline solution.

Group 2: cattle inoculated with urine, kidney, skin, bone marrow, buffy coat, cervical/popliteal lymph nodes, mesenteric lymph node, thymus, spleen, liver, salivary glands, peripheral nerve, skeletal muscle, spinal cord, caudal medulla or spinal cord/caudal medulla pool from orally BSE challenged cattle collected at various times post inoculation [16].

Group 3: cattle inoculated with tonsil [collected at 6, 10 (n = 4), 18, 26 mpi], distal ileum (collected at 26 and 32 mpi), caudal medulla (26 mpi), caudal medulla/spinal cord pool (26 mpi), spinal cord (26 mpi), peripheral nerve (32 mpi), nictitating membrane [n = 4 (receiving a pool of nictitating membrane from BSE field cases)].

Group 4: cattle inoculated with nictitating membrane (n = 1 [20], receiving a pool of nictitating membrane from BSE field cases), tonsil (n = 1, 10 mpi), distal ileum (6, 10, 18 mpi), caudal medulla/spinal cord pool (32 mpi).

The number of assessments (examinations and observations) per group are given in Table 1.

The Fisher's exact test (GraphPad Prism for Windows version 5. GraphPad Software, San Diego, USA) was used to compare clinical signs displayed at the last examination prior to cull. For behavioural observations, the last three observation periods prior to cull were taken into consideration to determine absence or presence of a sign. The null hypothesis was that clinical signs between controls and BSE-positive cattle and between controls and BSE-negative cattle inoculated with BSE brainstem homogenate or with tissue from BSE-challenged cattle are similar. The Fisher's exact test was also used to compare signs at pre-cull examinations between orally dosed BSE cases with and without vacuolar changes at the obex to test the null hypothesis that clinical signs in BSE cases with PrP^d ^and vacuolation in the brain are similar to BSE cases that have PrP^d ^but minimal/no vacuolation in the brain. The null hypothesis was rejected if *P*<0.05 for a particular sign associated with BSE.

Survival analysis [29] was used to test the null hypothesis that the frequencies of occurrence of clinical signs between controls and BSE-positive cattle and between controls and BSE-negative cattle inoculated with BSE brainstem homogenate or with tissue from BSE-challenged cattle are similar. The failure event was the observation of a particular sign in an animal and the survival time was measured from challenge to the first occurrence and between subsequent occurrences. It was assumed that a particular sign, if it was caused by BSE, should be displayed consistently at subsequent assessments. Statistical software (Stata/IC 10.0 for Windows, 2007. StatCorp LP, College Station, USA) was used to fit Cox proportional hazards models with multiple failures. Robust standard errors were specified to allow for the clustering by animal and the Efron method was used for handling ties in the calculation of the partial likelihood. When the Wald chi square test for the model indicated overall differences among the treatment groups of animals, specific contrasts were tested using further Wald tests. The significance level of the *P*-value was corrected for multiple comparisons (0.05/k where k equals the number of comparisons). The null hypothesis was rejected if *P*<0.0125 (study 1: comparison of BSE-positive cattle dosed with 100 g, BSE-positive cattle dosed with 1 g, BSE-negative cattle dosed with 100 g and BSE-negative cattle dosed with 1 g of BSE brainstem with control cattle) and *P*<0.0167 (study 2: comparison of group 4 cattle, group 3 cattle and group 2 cattle with group 1 control cattle) for a particular sign associated with BSE.

## Results

### Postmortem diagnosis

Of the postmortem tests used for BSE diagnosis [13,21], IHC for PrP^d ^with antibody R145 on sections of the medulla at the obex was, as previously indicated, the most sensitive confirmatory method [23]. Previously examined additional immunolabelled sections of brainstem (study 1) [23], or the additional immunolabelled sections of brainstem and rostral brain regions from all cattle in study 2, did not alter the diagnosis based solely on examination of medulla at the obex. Furthermore, examination of additional brain regions did not provide evidence of other neurological disease that might have accounted for clinical signs.

### Suspect BSE cases based on signs displayed on pre-cull examinations

The number of cattle, according to study and experimental group, that at the last examination prior to cull displayed signs associated with BSE in at least two of the categories "behavioural changes", "sensory changes" and "locomotor changes" is given in Table 1.

Examples of the signs observed in two cattle of study 2 are presented as Additional file 2: CN1150 (suspect-BSE positive), a confirmed BSE case that displayed behavioural, sensory and locomotor changes, and Additional file 3: CP1503 (suspect-BSE negative), an unconfirmed BSE suspect that displayed behavioural and locomotor abnormalities.

### Clinical signs assessed by examinations and observations prior to cull

Table 2 compares the clinical signs at the last examination prior to cull in BSE cases from study 1 confirmed on detection of PrP^d ^at the obex according to the occurrence of vacuolar changes. Clinical signs were generally more frequently recorded in cases with vacuolar changes at the obex, which was statistically significant for six signs or sign combinations. See Additional file 4: 143 (BSE positive-IHC) and Additional file 5: 139 (BSE positive-HP) to compare the severity of displayed clinical signs between two BSE cases, one of which (animal 143) had equivocal vacuolar changes in the medulla at the obex.

**Table 2:** Signs at the pre-cull examination in BSE cases confirmed on postmortem diagnosis in study 1 and grouped by the occurrence/severity of vacuolar changes in the brainstem

	Vacuolation (N = 21)	No or minimal vacuolation (N = 9)	*P *value
	**Present/N**^**a **^**(%)**	**Present/N**^**a **^**(%)**	

***Behaviour***			

Nervous or apprehensive in corridor	15/21 (71.4%)	5/9 (55.6%)	0.431

Nervous or head shy in crush	11/21 (52.4%)	2/9 (22.2%)	0.229

Crush refused or entered reluctantly	5/21 (23.8%)	2/9 (22.2%)	1.000

Obstacle refused or not approached	3/18 (16.7%)	1/9 (11.1%)	1.000

Kicked out - free	3/21 (14.3%)	0/9 (0%)	0.535

Yawning	3/21 (14.3%)	1/9 (11.1%)	1.000

Vocal - free	1/21 (4.8%)	1/9 (11.1%)	0.517

Teeth grinding	1/21 (4.8%)	0/9 (0%)	1.000

***Sensation***			

Clipboard test abnormal ≥1	14/18 (77.8%)	1/6 (16.7%)	0.015**

Over-reactivity to sound ≥1	14/21 (66.7%)	2/9 (22.2%)	0.046*

Flinch/startle free once	13/21 (61.9%)	3/9 (33.3%)	0.236

Tests of over-reactivity abnormal once/2 tests	13/21 (61.9%)	1/9 (11.1%)	0.017**

Flinch or startle free more than once	12/21 (57.1%)	2/9 (22.2%)	0.118

Head tests - nervous	12/21 (57.1%)	0/9 (0%)	0.004***

Clipboard test abnormal >2	9/18 (50.0%)	0/6 (0%)	0.052

Flash test abnormal ≥1	10/20 (50.0%)	0/8 (0%)	0.025*

Tests of over-reactivity abnormal x3/2 tests	8/21 (38.1%)	0/9 (0%)	0.067

Head restraint - nervous	1/21 (4.8%)	0/9 (0%)	1.000

Over-reactivity to sound >2	7/21 (33.3%)	2/9 (22.2%)	0.681

Exaggerated menace response	5/16 (31.3%)	0/9 (0%)	0.286

Flash test abnormal >2	4/20 (20.0%)	0/8 (0%)	0.295

Neck prick - nervous	8/21 (38.1%)	0/9 (0%)	1.000

Stick test abnormal	0/21 (0%)	0/9 (0%)	1.000

***Movement***			

Ataxia or hypermetria	14/20 (70.0%)	2/9 (22.2%)	0.041*

Tremor	15/21 (71.4%)	4/9 (44.4%)	0.225

^a ^The total number of animals varied because not all signs were assessed in all animals. Excluded are the signs "last in crush" and "nervous entering the milking parlour" because of the limited assessment of this sign in one or both groups.

*P *values are expressed quantitatively; values below 0.05 were considered significant and marked * (*P *= 0.02-0.05), ** (*P *= 0.01-0.02) or *** (*P *= 0-0.01).

The clinical signs in all cattle from both source studies grouped according to diagnosis and dose or inoculum are given in Table 3 (signs at the last examination prior to cull) and Table 4 (signs at the last three observation periods prior to cull). There was generally a significant difference in the clinical signs expressed by BSE-positive animals compared to controls, with signs usually expressed more frequently in BSE-positive cattle. With eight exceptions, the frequency of expressed signs between controls and inoculated BSE-negative animals did not differ significantly. Exceptions were less frequent in controls but for refusal to approach or cross an obstacle, which was more frequent in controls than BSE-negative, inoculated cattle in study 2. See Additional file 6: 10 (control) and Additional file 7: 138 (BSE negative-HP&IHC) as example of the nervous behaviour and startle responses that were comparatively less frequently exhibited in BSE-free controls (e.g. animal 10) than BSE-infected cattle that were not confirmed by postmortem tests (e.g. animal 138).

**Table 3:** Relative frequency of clinical signs expressed at the pre-cull examination compared to control cattle

Study	1	1	1	2	2	2
**Groups**	**1 g & 100 g**	**1 g**	**100 g**	**4**	**3**	**2**

**Postmortem BSE diagnosis**	**Pos**	**Neg**	**Neg**	**Pos**	**Neg**	**Neg**

***Behaviour***						

Crush refused or entered reluctantly	↑↑↑		↑↑↑			

Nervous or apprehensive in corridor	↑↑↑		↑↑	↑↑		

Obstacle refused or not approached	↑				↓↓	↓↓

Nervous entering milking parlour	↑			---	---	---

Yawning	↑					

Kicked out during examination	↑					

***Sensation***						

Startle repeatedly during examination	↑↑↑	↑↑↑	↑↑↑			

Startle once during examination	↑↑↑		↑			

Clipboard test abnormal once	↑↑↑					

Clipboard test abnormal >2	↑↑↑					

Over-reactivity to sound ≥1	↑↑↑					

Over-reactivity present once/2 tests	↑↑↑					

Over-reactivity present >2/2 tests	↑↑↑					

Over-reactivity to sound >2	↑↑					

Flash test abnormal ≥1	↑					

Flash test abnormal >2	↑					

Exaggerated menace	↑					

Head tests nervous				↑↑		

Stick test: kicking				↑		

***Movement***						

Tremor	↑↑↑			↑		

Ataxia or hypermetria	↑↑↑					

Groups were compared with undosed controls (study 1) or saline-inoculated controls (study 2). The BSE-positive animals dosed with 1 g and 100 g in study 1 were combined to increase the sample size.

(↑) increased frequency of sign; (↓) decreased frequency of sign compared to control cattle, with *P*<0.001 (three arrows), *P *= 0.001-0.01 (two arrows) and *P*>0.01 and <0.05 (one arrow). Blank cell: not significant (*P*>0.05) or no *P*-value given because of absence of this sign in both groups.

Only signs that were significantly differently displayed compared to controls are listed.

**Table 4:** Relative frequency of clinical signs expressed at the last three observation periods prior to cull compared to control cattle

Study	1	1	2
**Groups**	**1 g & 100 g**	**1 g**	**4**

**Postmortem BSE diagnosis**	**Pos**	**Neg**	**Pos**

***Behaviour***			

Apprehension	↑↑↑		↑↑

Unusual asymmetrical ear movements	↑↑↑		↑

Grooming self	↑		↑↑

Nose licking	↑		

Snorting	↑		

Sneezing	↑		

Head toss			↑

***Sensation***			

Hyperaesthesia	↑↑↑	↑	↑

Abnormal hand approach/clipboard test	↑↑↑		↑

Startle at movement	↑↑↑		

Startle at movement incl. hand approach/clipboard test	↑↑↑	↑	↑

Startle at noise	↑		

***Posture***			

Abnormal head carriage		↑	

Groups were compared with undosed controls (study 1) or saline-inoculated controls (study 2). The BSE-positive animals dosed with 1 g and 100 g in study 1 were combined to increase the sample size.

An arrow (↑) indicates that the sign or sign combination is more frequent than in control cattle, with *P*<0.001 (↑↑↑), *P *= 0.001-0.01 (↑↑) and *P*>0.01 and <0.05 (↑). Blank cell: not significant (*P*>0.05) or no *P*-value given because of absence of this sign in both groups.

Only signs that were significantly differently displayed compared to controls are listed. Not shown are the comparisons of signs between controls and BSE-negative cattle dosed with 100 g of BSE brainstem (study 1) and between controls and BSE-negative cattle in groups 2 and 3 (study 2) because there was no significant difference for any of the signs.

Signs assessed by clinical examinations that yielded no significant differences between any of the groups were "nervous on head restraint", "last animal in crush", "nervous on neck prick", "nervous or head shy in crush", "teeth grinding" and "vocal when free". Signs assessed by weekly observations with a similar outcome were "standing idle & not approached", "head rubbing", "flank licking", "nose wrinkling", "teeth grinding", "tremor", "muscle fasciculations", "vocalisation", "flehmen", "grooming others", "head toss with snort", "BSE series of events", "yawning", "coughing", "butting others" and "being butted", "mounting others" and "being mounted", "scratching" and "tongue playing or rolling".

There were also no significant differences for any of the signs assessed by observations between controls (group 1) and BSE-negative cattle inoculated with various tissues associated with or without infectivity (groups 2 and 3) in study 2.

### Clinical signs assessed by examinations and observations over time

Signs that were significantly differently expressed compared to control cattle are given in Table 5 (signs assessed by examinations) and table 6 (signs assessed by observations). No significant differences were found for the signs "last animal in crush", "obstacle refused or not approached", "exaggerated menace response" or "teeth grinding", which were assessed by examinations, and "yawning", "flehmen", "teeth grinding", "head toss with snort", "being butted", "butting others", "scratching" or "tongue playing/rolling", assessed by observations. For several signs, the incidence rate in animals of a study was too low to calculate a *P*-value because the sign was rarely expressed, such as "nervousness during head restraint or during head tests" in cattle of study 1. If significant differences were found between controls and other groups, the hazard rate, i.e. the frequency of a sign displayed over a time interval, and the total number of signs were generally greater in animals with confirmed BSE than BSE-negative, inoculated animals. Signs with a significantly lower hazard rate compared to controls were predominantly displayed by BSE-negative, inoculated animals and mainly comprised signs assessed by observations.

**Table 5:** Relative frequency of clinical signs at neurological examinations over time post inoculation compared to control cattle

Study	1	1	1	1	2	2
**Groups**	**1 g**	**100 g**	**1 g**	**100 g**	**4**	**2**

**Postmortem BSE diagnosis**	**Pos**	**Pos**	**Neg**	**Neg**	**Pos**	**Neg**

***Behaviour***						

Nervous or apprehensive in corridor	↑↑↑	↑↑↑		↑↑↑	↑↑↑	

Nervous entering milking parlour	↑↑↑			↑↑↑	---	---

Kicked out during examination	↑↑	↑↑		LI	LI	LI

Nervous/head shy in crush	↑	↑↑↑			↑↑↑	

Vocal free				↑↑↑		

Crush refused or entered reluctantly	LI	LI	LI	LI	↑	

Yawning						↑↑↑

***Sensation***						

Startle during examination once	↑↑↑	↑↑↑		↑↑↑	↑↑↑	

Startle during examination repeatedly	↑↑↑	↑↑↑	↑↑	↑↑↑	↑↑↑	

Clipboard test abnormal ≥1	↑↑↑	↑↑↑			↑↑	

Clipboard test abnormal >2	↑↑↑	↑↑↑				

Over-reactivity to sound once		↑↑↑		↑↑↑		

Over-reactivity present once/2 tests		↑↑↑		↑↑	↑↑	

Over-reactivity present >2/2 tests		↑↑↑			↑↑↑	

Over-reactivity to sound >2		↑↑↑			↑↑	

Flash test abnormal >2		↑↑↑			LI	

Flash test abnormal ≥1		↑↑↑				↓

Stick test: kicking	LI	LI	LI	LI	↑↑↑	

Head tests nervous	LI	LI	LI	LI	↑↑	

Head restraint nervous	LI	LI	LI	LI		↓

***Movement***						

Tremor	↑↑	↑↑↑		↑↑	↑↑↑	

Ataxia or hypermetria	↑↑	↑↑↑			↑↑↑	

Groups were compared with undosed controls (study 1) or saline-inoculated controls (study 2).

↑= greater/↓ = lower hazard rate than controls, with *P*<0.001 (three arrows), *P *= 0.001-0.01 (two arrows) and *P*>0.01 and <0.0125 (four comparisons) or <0.0167 (three comparisons) (one arrow). Blank cell: no significant difference; low incidence (LI): signs too rare in at least one of the groups to calculate a *P*-value.

Only signs that were significantly differently displayed compared to controls are listed. Groups are omitted if none of the signs were significantly different compared to controls.

Not shown is the comparison of signs between controls and cattle inoculated with tissue of expected infectivity (group 3, study 2) because there was no significant difference for any of the signs.

**Table 6:** Relative frequency of clinical signs at observations over time post inoculation compared to control cattle

Study	1	1	1	1	2	2	2
**Groups**	**100 g**	**1 g**	**1 g**	**100 g**	**4**	**3**	**2**

**Postmortem BSE diagnosis**	**Pos**	**Pos**	**Neg**	**Neg**	**Pos**	**Neg**	**Neg**

***Behaviour***							

Flank licking	↑↑↑			↑↑	↓↓↓	↓↓↓	↓↓↓

Unusual asymmetrical ear movements	↑↑↑				↑↑		

Vocalisation	↑↑						

Head toss	↑↑						

Snorting	↑↑	↓↓↓					

Nose licking	↑↑						↓↓↓

Grooming self		↑↑	↑↑				

Apprehension		↑↑			↑↑↑		

BSE series of events		↓↓↓			↑↑		↑

Grooming others			↑↑				↑↑

Standing idle & not approached			↓↓↓	↓↓↓	↑↑↑		

Mounting				↑↑	↑↑↑	↑↑	↑↑↑

Sneezing					↑↑		

Being mounted					↑		

Head rubbing						↓↓↓	

Nose wrinkling							↓↓↓

Coughing							↓↓↓

***Sensation***							

Startle at movement including hand approach/clipboard test	↑↑↑	↑↑↑			↑↑↑		

Hyperaesthesia	↑↑↑	↓↓			↑↑↑		

Startle at movement	↑↑↑			↑	↑↑↑	↑↑↑	

Startle at noise	↑↑				↑↑↑		

Abnormal hand approach/clipboard test					↑↑		

***Movement and posture***							

Muscle fasciculations	↑↑		↑↑↑	↑↑			

Tremor					↑↑		

Abnormal head carriage							↓

Groups were compared with undosed controls (study 1) and saline-inoculated controls (study 2).

↑= greater/↓ = lower hazard rate than controls, with *P*<0.001 (three arrows), *P *= 0.001-0.01 (two arrows) and *P*>0.01 and <0.0125 (four comparisons) or <0.0167 (three comparisons) (one arrow). Blank cell: no significant difference.

Only signs that were significantly differently displayed compared to controls are listed.

## Discussion

BSE is characterised by a combination of changes in behaviour, sensation and movement, although the contributory signs that define these categories can be variable [3,30]. It has been shown that BSE should be considered in cattle that display a combination of apprehension, over-reactivity (to touch or sound) and ataxia, since at least one of these signs featured in 97% of 17,154 cases [3]. Because of the experimental designs of the two source studies in the present investigation these criteria are not applicable to the entire population of animals studied. For example, using the approach for at least two of the signs apprehension or nervousness, over-reactivity (as evaluated by tests of over-reactivity including repeated spontaneous startle responses) and ataxia or hypermetria, not all experimentally inoculated, BSE-confirmed cattle were clinical suspects under these criteria. This was to be expected in study 1 where animals were culled at pre-defined time points post challenge and therefore some cattle were in the early stages of disease, or not infected, and thus would not necessarily display the full spectrum or any of the signs associated with BSE. In contrast, the cattle in study 2 were culled at clinical end-point when the signs were convincing enough to suspect BSE, unless, in some cases, concerns for staff and animal health and welfare due to the animal's behaviour precluded monitoring further progression of signs, such as ataxia. The selection of criteria to define a clinical suspect as part of this investigation may explain why some BSE-positive cases were not identified as clinical suspects: for example, one BSE case inoculated with distal ileum from orally dosed cattle culled at 10 mpi developed only over-reactivity (marked aversion to touch in combination with frequent startle reaction), which was convincing enough to suspect BSE, although neither nervousness nor ataxia were displayed.

Conversely, 17 cattle of study 2 that were inoculated with tissue from BSE-infected cattle were not confirmed to have BSE based on postmortem tests and no alternative diagnosis was established but they did display the sign combination for a clinical BSE suspect. While it is possible that other conditions affecting the CNS or other organ systems might cause behavioural, sensory or locomotor abnormalities, which in combination are suggestive of BSE, no such circumstances occurred in controls. Similar observations have been made in a separate study in casualty slaughtered cattle [31].

The design, particularly group sizes, of the source studies presented difficulty in analysing the clinical signs with regards to the choice of statistical tests. Two different methods were chosen to compare the frequency of expressed clinical signs, either taking into account repeated observations or using only the last assessment prior to cull. BSE is a slowly progressive disease and signs are displayed more frequently, more consistently and more severely as the disease progresses, which makes Cox regression survival analysis an appropriate method. However, in study 1 cattle were culled at a predetermined time point post challenge or, in both studies, at a predetermined end point when the development of BSE was not expected in surviving animals. Therefore, some of the animals may have been at an early stage of the disease when signs associated with BSE were observed only shortly before culling and not yet repeatedly. In these cases, the Fisher's exact test was more applicable, which only considered the occurrence of a sign prior to cull. Regardless of the statistical method, the results confirmed, as expected, that the selected signs associated with BSE occurred more frequently in groups with diagnostic changes of vacuolation and/or PrP^d ^accumulation.

The histopathological diagnosis based on vacuolar changes is less sensitive than detection of PrP^d ^by IHC, which has been demonstrated in experimental BSE [13,18,32], since, in general, histopathological changes are apparent later in the course of the disease than detection of PrP^d^. The more frequent occurrence of locomotor and sensory signs in cattle with vacuolar changes compared to those with no or equivocal vacuolation (Table 2) suggests that vacuolation coincides with a more advanced clinical stage. This is in agreement with the findings in a Swiss study, where clinical suspect BSE cases had more severe spongiform changes in the brain than BSE cases slaughtered as apparently healthy cattle [33].

The great variation in the group sizes meant that for some signs statistical analysis was compromised because the number of animals per group was too small. It was considered appropriate to combine BSE-positive animals dosed with 1 g and 100 g to increase the sample size for comparison of signs prior to cull although this was less appropriate for the analysis of signs over time, which may be dependent on the dose. It has been demonstrated in cattle orally challenged with BSE that the incubation period followed a lognormal distribution, with decreasing mean as dose increased [13], and thus a similar effect of dose on onset of the first clinical signs and possibly the disease duration is expected. In study 2 in particular, the number of animals in some groups was considerably greater than in others, ranging from 10 to 240. As a result of the disproportionate group sizes, the differences in the signs displayed by BSE-negative cattle inoculated with tissues from cattle orally dosed with BSE-affected brain and controls were usually not significant, or signs were even less frequently observed over a time period than in controls. In these instances, comparison of signs on a case-to-case basis may be more appropriate, like the selection of clinical markers to identify clinical suspects as demonstrated in Table 1.

Although the signs used for comparison of the different groups of cattle in different source studies consisted of behavioural, sensory and locomotor signs associated with BSE [3,24,25], many of these signs are unspecific, or not characteristic of a neurological disease and may also be displayed to some degree in healthy cattle. This may explain why some of the signs were more frequent in BSE-positive cattle of one study but not in the other. For example, "BSE series of events", which were defined as behavioural signs in combination with head tossing or nose licking, all of which were associated with BSE [25], were usually significantly more frequent in BSE-positive cattle of study 2 but were unexpectedly significantly less frequent in BSE-positive cattle dosed with 1 g compared to controls of study 1. Other signs that may be neurogenic, such as tremor, may occur physiologically in anxious animals or may be displayed as hind limb tremor in animals with spastic syndrome, which occurred in some animals but was not considered to be associated with BSE (data not shown). To simplify the analysis, the absence or presence of tremor was assessed regardless of location and underlying condition. In most cases, in particular when the tremor was not confined to the head, it was impossible to determine the cause based on a clinical examination and to differentiate a potential physiological tremor or a tremor caused by other intercurrent diseases from an identical tremor displayed by BSE-affected cattle. Furthermore, the finding that significant differences were not consistently present between BSE-positive cattle and controls, for example in the display of nervousness on head tests or head restraint, made it difficult to interpret the significance of these signs. This may be partly explained by the different treatment of animals between studies, such as regular blood sampling, which was done only in cattle of study 1. We also cannot rule out that the route of inoculation (oral or intracerebral) has an effect on the display of particular signs since the route of infection may result in minor differences in the severity of vacuolar changes [32,34,35] although cattle with BSE have generally presented with changes in behaviour, sensation and locomotion irrespective of the route [32,36].

A predominant sign of BSE is over-reactivity to external stimuli, which has been observed in 75-98% of confirmed clinical field cases [30]. Its presence was so characteristic in BSE that tests of over-reactivity have been refined successfully to diagnose BSE [24], to predict cases among BSE suspects prior to postmortem test confirmation [5] and to screen casualty slaughter cattle for BSE [31]. Startle responses are also observed frequently in patients suffering from sporadic Creutzfeldt-Jakob disease [37,38]. Indeed, startle responses to tests of over-reactivity and apparently spontaneous startle, occurring during examinations or observed on weekly observations, were consistently more frequent in BSE-positive animals compared to controls and this was often statistically significant. Interestingly, the repeated display of startle responses was also significantly more frequent in cattle orally dosed with 1 g and 100 g of BSE brainstem in study 1 despite a negative postmortem test result, regardless of the statistical method used. Similarly, cattle inoculated with tissue from BSE-challenged cattle (study 2, group 3) startled to the observer's movement significantly more often than control cattle when signs over time were compared. Nervousness or apprehension in the corridor and tremor were also significantly more frequently displayed in BSE-negative cattle orally dosed with 100 g using the Cox regression analysis. In the absence of histopathological changes in the brains of these cattle and the exclusion of metabolic diseases by regular blood biochemical analysis of selected cattle in the study (data not shown), it is possible that the signs were actually caused by BSE. There are some precedents for this in other species, such as mice [39] and pigs [40] inoculated with the BSE agent and goats naturally infected with scrapie [41], which presented with signs suggestive of a TSE despite failure to detect PrP^d ^in the brain. In a previous study, of the animals in the present study 1, which sought to predict the relationship between detection of PrP^d ^and incubation period according to dose, the clinical criteria were based on the combination of clinical signs consistent with the "definite" clinical diagnosis of BSE which was considered equivalent to reporting of the animal as a suspect in the field [13]. This approach includes the use of classifications of possible and probable signs of BSE [7] where "possible" and "probable" indicate a potential earlier clinical stage based on the relative frequency and severity of signs or sign combinations. Using this approach, it has been shown that some animals in both of the present studies may display possible or probable signs in the absence of a positive postmortem test result [14,21].

Improvement of postmortem test protocols over time has accounted for increased sensitivity of the diagnosis of BSE cases in an experimental oral exposure cattle study [13,42]. Also, studies utilising brain tissue from selected orally dosed, postmortem test unconfirmed cattle in study 1, have demonstrated the presence of infectivity by assay in transgenic mice overexpressing bovine PrP [26] and the presence of PrP^res ^by PMCA [43]. There are therefore clear precedents for the prudent application of additional transmissibility, molecular and neuropathological assessments of clinically healthy animals inoculated with a TSE agent to exclude the possibility of subclinical prion infection, which has been often demonstrated in rodent studies [44]. The possible occurrence of clinical disease in cattle unconfirmed by current postmortem tests equally warrants further investigation.

## Conclusions

Selected signs associated with BSE were more frequently displayed in pathologically confirmed experimental BSE cases compared to un-inoculated controls and, among confirmed cases, signs were more frequently expressed in cases with vacuolar pathology, indicative of a more advanced stage of disease. However, certain behavioural and sensory signs associated with BSE or a combination of behavioural, sensory or locomotor signs suggestive of BSE were also found more frequently in BSE inoculated cattle that were not confirmed by postmortem examinations. It is suggested that the additional techniques of tissue bioassay in transgenic mice overexpressing bovine PrP and PMCA are required to determine the possible significance of this apparent insensitivity of the diagnostic postmortem examinations.

## List of abbreviations

BSE: Bovine spongiform encephalopathy; CNS: Central nervous system; ELISA: Enzyme-linked immunosorbent assay; HP: Histopathological examination; IHC: Immunohistochemical examination; MBM: Meat and bone meal; mpi: Months post inoculation; PMCA: Protein misfolding cyclic amplification; PrP^d^: Disease-associated prion protein; PrP^res^: Proteinase-resistant fragment of PrP^d^; TSE: Transmissible spongiform encephalopathy; UK: United Kingdom; VLA: Veterinary Laboratories Agency; WB: Western immunoblot

## Authors' contributions

TK drafted the manuscript, carried out the vast majority of the clinical examinations and - supported by SVW - interpreted the clinical data. AS and GEB contributed to the clinical data acquisition and analysis. ARS supervised statistical calculations and carried out the survival analysis. GAHW and MMS were responsible for the neuropathological diagnosis, MJS for the Western blot and AW for the ELISA. The cattle projects were managed by SACH.

All authors read and approved the final manuscript.

## Supplementary Material

Additional file 1 **Assessed signs**. Clinical signs used for comparison and definition of the sign. Click here for file

Additional file 2 **CN1150 (suspect-BSE positive)**. Steer, examined prior to cull at 47 months after intracerebral inoculation with a pool of palatine tonsil collected from cattle orally exposed to BSE affected brainstem and killed 10 mpi. The steer does not over-react to testing of facial sensation although it retracts its head in the yoke of the crush and later becomes agitated. A mild spontaneous startle occurs during locomotion after the steer crosses the hose on the floor. Ataxia is evident when it jumps over the hose and approaches its pen mates (released into the corridor because of the steer's reluctance to walk). Note the steer's apprehension when close to the camera at the end of the clip. It also shows poor bodily condition. This steer was positive for BSE by postmortem tests with vacuolar changes in the obex. Click here for file

Additional file 3 **CP1503 (suspect-BSE negative)**. Steer, examined prior to cull at 87 months after intracerebral inoculation with a pool of spleens collected from cattle orally exposed to BSE affected brainstem and killed 18 mpi (study 2). This steer is slightly over-reactive to cranial nerve testing. It also over-reacts to touch on the neck (recorded as "nervous on neck prick"). It does not over-react to sudden flash light but a head tremor is evident during the procedure. The steer displays marked hind limb hypermetria, which is most pronounced as it reaches the end of the corridor. The steer also appears apprehensive in the corridor, as indicated by hesitation in passing the examiner and a very alert facial expression with ear movements when close to the camera. Note also the head and neck tremor when the animal stands near the camera at the end of the clip. This steer was negative for BSE by postmortem tests. Histopathologically, there was no evidence of a cerebellar lesion which may have produced hypermetria and head tremor. Click here for file

Additional file 4 **143 (BSE positive-IHC)**. Steer, examined prior to cull at 42 months after oral dosing with 100 g of BSE affected brainstem homogenate (study 1). This steer does not over-react to cranial nerve testing but repeatedly startles at a sudden metallic sound (recorded as "repeated over-reactivity to sound"). It appears apprehensive in the corridor, circles on the spot and startles when it is encouraged to move away from the gate by hand clapping. The histopathological diagnosis was inconclusive for BSE (minimal vacuolation in the obex) but BSE was confirmed by other postmortem tests. Click here for file

Additional file 5 **139 (BSE positive-HP)**. Steer, examined prior to cull at 39 months after oral dosing with 100 g of BSE affected brainstem homogenate (study 1). This steer is apprehensive in the corridor and very reluctant to approach the crush, seemingly afraid of the channel and later the wet patch on the floor. Note the spontaneous startle while approaching the crush. Its reaction to menace response testing is exaggerated and it over-reacts to facial stimuli with repeated rapid, forceful upwards head movements (head bobbing, recorded as 'nervous on head tests'). Exposure to a flash of light elicits startle, even on repeated tests. This steer was positive for BSE by postmortem tests with vacuolar changes in the obex. Click here for file

Additional file 6 **10 (control)**. Undosed control steer, examined prior to cull at 45 months of age. It over-reacts to facial stimuli, but not menace response testing. The head tossing activity does not resemble the head bobbing movements of steer 139. During locomotion the steer appears calm and stops to explore various objects along its path. This steer was negative for BSE by postmortem tests. Click here for file

Additional file 7 **138 (BSE negative-HP&IHC)**. Steer, examined prior to cull at 42 months after oral dosing with 100 g of BSE brainstem homogenate (study 1). The steer's behaviour during cranial nerve testing is unremarkable. It appears apprehensive in the corridor, particularly on approaching the channel and drain and the hose across the floor. Trembling of the rump muscles is visible from behind when the animal stops in front of the channel before crossing it. Note also the mild startle when the examiner walks in front of the steer whilst it is exploring the channel. This steer was negative for BSE by postmortem tests upon examination of the brain. In a separate study, PrP^d ^was found in the lymphoid follicles of the ileum of this steer, which suggests that it was infected with the BSE agent (GAH Wells and M Stack, unpublished observation). Click here for file

## References

[B1] Wells GA Scott AC Johnson CT Gunning RF Hancock RD Jeffrey M Dawson M Bradley R A novel progressive spongiform encephalopathy in cattle Vet Rec 1987 121 419 420 10.1136/vr.121.18.419 3424605

[B2] Wilesmith JW Wells GA Cranwell MP Ryan JB Bovine spongiform encephalopathy: epidemiological studies Vet Rec 1988 123 638 644 3218047

[B3] Wilesmith JW Hoinville LJ Ryan JB Sayers AR Bovine spongiform encephalopathy: aspects of the clinical picture and analyses of possible changes 1986-1990 Vet Rec 1992 130 197 201 10.1136/vr.130.10.197 1509649

[B4] Braun U Pusterla N Schicker E Bovine spongiform encephalopathy: Diagnostic approach and clinical findings Comp Cont Educ Pract Vet 1998 20 S270 S278

[B5] Konold T Bone G Ryder S Hawkins SAC Courtin F Berthelin-Baker C Clinical findings in 78 suspected cases of bovine spongiform encephalopathy in Great Britain Vet Rec 2004 155 659 666 10.1136/vr.155.21.659 15581140

[B6] Braun U Hauri S Kihm U Ehrensperger F Evaluation des BSE-Checks bei weiteren 53 Kühen mit BSE [Evaluation of a clinical examination specifically designed to detect bovine spongiform encephalopathy] Tierärztl Prax Ausg Gros Nutz 2007 35 111 116

[B7] Wells GAH Hawkins SAC Lehmann S, Grassi J Animal models of transmissible spongiform encephalopathies: Experimental infection, observation and tissue collection Techniques in prion research 2004 Basel: Birkhäuser Verlag 37 71

[B8] Wells GAH Hancock RD Cooley WA Richards MS Higgins RJ David GP Bovine spongiform encephalopathy: Diagnostic significance of vacuolar changes in selected nuclei of the medulla oblongata Vet Rec 1989 125 521 524 10.1136/vr.125.21.521 2512718

[B9] Scott AC Wells GAH Stack MJ White H Dawson M Bovine spongiform encephalopathy: detection and quantitation of fibrils, fibril Protein (PrP) and vacuolation in brain Vet Microbiol 1990 23 295 304 10.1016/0378-1135(90)90160-W 1976286

[B10] Prusiner SB Fuzi M Scott M Serban D Serban H Taraboulos A Gabriel JM Wells GAH Wilesmith JW Bradley R DeArmond SJ Kristensson K Immunological and molecular biologic studies of prion proteins in bovine spongiform encephalopathy J Infect Dis 1993 167 602 613 8440932 10.1093/infdis/167.3.602

[B11] Gavier-Widén D Stack MJ Baron T Balachandran A Simmons M Diagnosis of transmissible spongiform encephalopathies in animals: a review J Vet Diagn Invest 2005 17 509 527 16475509 10.1177/104063870501700601

[B12] Office International des Epizooties OIE Biological Standards Commission Bovine spongiform encephalopathy OIE manual of diagnostic tests and vaccines for terrestrial animals (mammals, birds and bees) 2008 6 Paris: Office International des Epizooties 671 682

[B13] Arnold ME Ryan JBM Konold T Simmons MM Spencer YI Wear A Chaplin M Stack M Czub S Mueller R Webb PR Davis A Spiropoulos J Holdaway J Hawkins SAC Austin AR Wells GAH Estimating the temporal relationship between PrPSc detection and incubation period in experimental bovine spongiform encephalopathy of cattle J Gen Virol 2007 88 3198 3208 10.1099/vir.0.82987-0 17947547

[B14] Masujin K Matthews D Wells GAH Mohri S Yokoyama T Prions in the peripheral nerves of bovine spongiform encephalopathy-affected cattle J Gen Virol 2007 88 1850 1858 10.1099/vir.0.82779-0 17485547

[B15] McGill IS Wells GAH Neuropathological findings in cattle with clinically suspect but histologically unconfirmed bovine spongiform encephalopathy (BSE) J Comp Pathol 1993 108 241 260 10.1016/S0021-9975(08)80288-5 8315053

[B16] Wells GAH Spiropoulos J Hawkins SAC Ryder SJ Pathogenesis of experimental bovine spongiform encephalopathy: preclinical infectivity in tonsil and observations on the distribution of lingual tonsil in slaughtered cattle Vet Rec 2005 156 401 407 15816193 10.1136/vr.156.13.401

[B17] Simmons MM Spiropoulos J Webb PR Spencer YI Czub S Mueller R Davis A Arnold ME Marsh S Hawkins SAC Cooper JA Konold T Wells GAH Experimental classical bovine spongiform encephalopathy: definition and progression of neural PrP immunolabeling in relation to diagnosis and disease controls Vet Pathol in press 10.1177/0300985810387072 21078883

[B18] Wells GAH Hawkins SAC Green RB Austin AR Dexter I Spencer YI Chaplin MJ Stack MJ Dawson M Preliminary observations on the pathogenesis of experimental bovine spongiform encephalopathy (BSE): an update Vet Rec 1998 142 103 106 10.1136/vr.142.5.103 9501384

[B19] Simmons MM Spiropoulos J Hawkins SA Bellworthy SJ Tongue SC Approaches to investigating transmission of spongiform encephalopathies in domestic animals using BSE as an example Vet Res 2008 39 34 10.1051/vetres:2008011 18284911

[B20] World Health Organization WHO guidelines on tissue infectivity distribution in transmissible spongiform encephalopathies 2006 Geneva: WHO Press

[B21] Sohn HJ Lee YH Green RB Spencer YI Hawkins SAC Stack MJ Konold T Wells GAH Matthews D Cho IS Joo YS Bone marrow infectivity in cattle exposed to the bovine spongiform encephalopathy agent Vet Rec 2009 164 272 273 10.1136/vr.164.9.272 19252214

[B22] Stack MJ Lehmann S, Grassi J Western immunoblotting techniques for the study of transmissible spongiform encephalopathies Techniques in prion research 2004 Basel: Birkhäuser Verlag 97 116

[B23] Arnold ME Ryan JBM Konold T Simmons MM Spencer YI Wear A Chaplin M Stack M Czub S Mueller R Webb PR Davis A Spiropoulos J Holdaway J Hawkins SAC Austin AR Wells GAH Estimating the temporal relationship between PrPSc detection and incubation period in experimental bovine spongiform encephalopathy of cattle J Gen Virol 2007 88 3198 3208 10.1099/vir.0.82987-0 17947547

[B24] Braun U Kihm U Pusterla N Schönmann M Klinischer Untersuchungsgang bei Verdacht auf bovine spongiforme Enzephalopathie (BSE) [Clinical examination upon suspicion of bovine spongiform encephalopathy (BSE)] Schweiz Arch Tierheilkd 1997 139 35 41 9312849

[B25] Austin AR Hawkins SAC Kelay NS Simmons MM Bradley R, Marchant B New observations on the clinical signs of BSE and scrapie Transmissible spongiform encephalopathies. Proceedings of a consultation on BSE with the Scientific Veterinary Committee of the Commission of the European Communities: 14-15 September 1993; Brussels 1994 Brussels: European Commission, Agriculture 277 287

[B26] Espinosa JC Morales M Castilla J Rogers M Torres JM Progression of prion infectivity in asymptomatic cattle after oral bovine spongiform encephalopathy challenge J Gen Virol 2007 88 1379 1383 10.1099/vir.0.82647-0 17374785

[B27] Hoffmann C Ziegler U Buschmann A Weber A Kupfer L Oelschlegel A Hammerschmidt B Groschup MH Prions spread via the autonomic nervous system from the gut to the central nervous system in cattle incubating bovine spongiform encephalopathy J Gen Virol 2007 88 1048 1055 10.1099/vir.0.82186-0 17325380

[B28] Terry LA Marsh S Ryder SJ Hawkins SAC Wells GAH Spencer YI Detection of disease-specific PrP in the distal ileum of cattle exposed orally to the agent of bovine spongiform encephalopathy Vet Rec 2003 152 387 392 10.1136/vr.152.13.387 12696704

[B29] Cleves MA Gould WW Gutierrez RG Marchenko YU Introduction to survival analysis using Stata 2008 College Station, USA: Stata Press

[B30] Schicker E Braun U Hörnlimann B Konold T Hörnlimann B, Riesner D, Kretzschmar H Clinical findings in bovine spongiform encephalopathy Prions in humans and animals 2006 Berlin: de Gruyter http://www.degruyter.de/cont/fb/na/detailEn.cfm?isbn=978-3-11-020017-1 full_text

[B31] Konold T Sivam SK Ryan J Gubbins S Laven R Howe MJ Analysis of clinical signs associated with bovine spongiform encephalopathy in casualty slaughter cattle Vet J 2006 171 438 444 10.1016/j.tvjl.2005.02.020 16624709

[B32] Wells GAH Konold T Arnold ME Austin AR Hawkins SAC Stack M Simmons MM Lee YH Gavier-Widén D Dawson M Wilesmith JW Bovine spongiform encephalopathy: the effect of oral exposure dose on attack rate and incubation period in cattle J Gen Virol 2007 88 1363 1373 10.1099/vir.0.82421-0 17374783

[B33] Sisó S Doherr MG Botteron C Fatzer R Zurbriggen A Vandevelde M Seuberlich T Neuropathological and molecular comparison between clinical and asymptomatic bovine spongiform encephalopathy cases Acta Neuropathol 2007 114 501 508 10.1007/s00401-007-0283-9 17763857

[B34] Konold T Lee YH Stack MJ Horrocks C Green RB Chaplin M Simmons MM Hawkins SA Lockey R Spiropoulos J Wilesmith JW Wells GA Different prion disease phenotypes result from inoculation of cattle with two temporally separated sources of sheep scrapie from Great Britain BMC Vet Res 2006 2 31 10.1186/1746-6148-2-31 17044917 PMC1636635

[B35] Wells GAH Simmons MM The essential lesion profile of bovine spongiform encephalopathy (BSE) in cattle is unaffected by breed or route of infection Neuropathol Appl Neurobiol 1996 22 453

[B36] Dawson M Wells GAH Parker BNJ Preliminary evidence of the experimental transmissibility of bovine spongiform encephalopathy to cattle Vet Rec 1990 126 112 113 2309405

[B37] Boesenberg C Schulz-Schaeffer WJ Meissner B Kallenberg K Bartl M Heinemann U Krasnianski A Stoeck K Varges D Windl O Kretzschmar HA Zerr I Clinical course in young patients with sporadic Creutzfeldt-Jakob disease Ann Neurol 2005 58 533 543 10.1002/ana.20568 16037975

[B38] Zerr I Poser S Clinical diagnosis and differential diagnosis of CJD and vCJD. With special emphasis on laboratory tests APMIS 2002 110 1 88 98 10.1034/j.1600-0463.2002.100111.x 12064260

[B39] Lasmézas CI Deslys JP Robain O Jaegly A Beringue V Peyrin JM Fournier JG Hauw JJ Rossier J Dormont D Transmission of the BSE agent to mice in the absence of detectable abnormal prion protein Science 1997 275 402 405 10.1126/science.275.5298.402 8994041

[B40] Konold T Spiropoulos J Chaplin MJ Thorne L Spencer YI Wells GAH Hawkins SAC Transmissibility studies of vacuolar changes in the rostral colliculus of pigs BMC Vet Res 2009 5 35 10.1186/1746-6148-5-35 19765298 PMC2761866

[B41] Konold T Bone GE Phelan LJ Simmons MM González L Sisó S Goldmann W Cawthraw S Hawkins SAC Monitoring of clinical signs in goats with transmissible spongiform encephalopathies BMC Vet Res 2010 6 13 10.1186/1746-6148-6-13 20202205 PMC2875668

[B42] Grassi J Comoy E Simon S Creminon C Frobert Y Trapmann S Schimmel H Hawkins SAC Moynagh J Deslys JR Wells GAH Rapid test for the preclinical postmortem diagnosis of BSE in central nervous system tissue Vet Rec 2001 149 577 582 10.1136/vr.149.19.577 11730165

[B43] Soto C Anderes L Suardi S Cardone F Castilla J Frossard MJ Peano S Saa P Limido L Carbonatto M Ironside J Torres JM Pocchiari M Tagliavini F Pre-symptomatic detection of prions by cyclic amplification of protein misfolding FEBS Lett 2005 579 638 10.1016/j.febslet.2004.12.035 15670821

[B44] Hill AF Collinge J Prion strains and species barriers Contrib Microbiol 2004 11 33 49 full_text 15077403 10.1159/000077061

