# Supplementary material for: Relationship between clinical signs and postmortem test status in cattle experimentally infected with the bovine spongiform encephalopathy agent
Source: BMC Vet Res. 2010 Dec 9;6:53. doi: 10.1186/1746-6148-6-53 (PMC3019182; doi:10.1186/1746-6148-6-53)
Supplement: Additional file 1 — Assessed signs. Clinical signs used for comparison and definition of the sign. [file 1746-6148-6-53-S1.DOC]

### 1. Signs assessed by clinical examinations

| **SIGN** | **DESCRIPTION** |
| --- | --- |
| ***Behaviour*** | |
| Crush refused or entered reluctantly | An animal was considered reluctant to enter the crush if it required forceful pushing (e.g. by two people). |
| Kicked out when free | Kicking out towards observing person. |
| Last in crush | Animals were kept in holding pen and could enter the crush in the order they chose; only evaluated in animals of study 1. |
| Nervous entering milking parlour | Marked hesitation or refusal to enter with tendency to panic or back off suddenly. Assessed only in male cattle of study 1, which were not used to entering the parlour. |
| Nervous or apprehensive in corridor | Runs away when approached, reluctant or refused to walk towards end of corridor, afraid of lines on floor. |
| Nervous or head shy in crush | Jumped back when approached for head restraint, twisting head or head down when faced, going down in crush when faced. |
| Obstacle refused or not approached | Different objects were chosen as obstacles, such as plastic bags, a hose or an iron bar. |
| Teeth grinding | Any teeth grinding during handling or when free. |
| Vocal – free | Any vocalisation when the animal was in the corridor. |
| Yawning | Any yawning regardless of stimulus. |
| ***Sensation*** | |
| Clipboard test abnormal | Abnormal response to a clipboard waved towards the animal (startle, flinch, charging, backing off or running away), either at least once or repeatedly more than twice. |
| Exaggerated menace | Flinch or startle, tossing or pulling back of head. |
| Flash test abnormal | Assessment of over-reactivity (startle, flinch) to flash light, either at least once or repeatedly more than twice. |
| Nervous on head restraint | Marked aversion to head restraint, e.g. head tossing, struggling. |
| Nervous on head tests | Marked aversion to head tests, i.e. assessment of the blink reflex or touching of the nose difficult to perform. |
| Nervous on neck prick | Marked aversion to pricking of the neck with forceps, e.g. head tossing or shaking. |
| Over-reactivity to sound | Abnormal response (startle, flinch) to either hand clap or bang test, either at least once or repeatedly more than twice. |
| Startle once or repeated startle during the examination | Any flinch or startle displayed in the corridor or when handled in the crush (spontaneous or unexpectedly to visual or auditory stimuli, such as moving in front of the animal or closing the crush door), which were not elicited by tests of over-reactivity. |
| Stick test abnormal | Any kicking when hind limbs are touched (soft or forceful) at least once. |
| Tests of over-reactivity | One abnormal response in at least two tests and – separately – at least three consecutive abnormal responses in at least two tests. Kicking with the hind limbs in response to the stick test, even if elicited only once, was always considered to be abnormal. |
| ***Movement*** | |
| Ataxia or hypermetria | Incoordination regardless whether fore or hind limbs were affected. |
| Tremors | Tremor of head, leg or whole body. |

### 2. Signs assessed by passive observations

| **SIGN** | **DESCRIPTION** |
| --- | --- |
| ***Behaviour*** | |
| Abnormal head carriage | Standing with head tilted to one side or with head raised or lowered. |
| Apprehension | Animal appears slightly nervous, holds back, yet may approach. |
| Being butted | Does not include head to head challenge. |
| Being mounted | Includes attempt, even if unsuccessful. |
| BSE series of events | Head tossing with nose licking or each event in combination with ear flapping, flank licking, head rubbing or teeth grinding. |
| Butting others | Does not include head to head challenge. |
| Coughing |  |
| Flehmen | Extension of the neck with nose wrinkling and raising the top lip exposing the gum (vomeronasal organ). |
| Grooming others | Licking parts of the body of another animal. |
| Grooming self | Licking parts of the body. |
| Head toss | Flicking or shaking of the head upwards or sideways. |
| Head toss with snort | Flicking or shaking of the head upwards or sideways, accompanied by snorting. |
| Licking flank | Flick of the head round to either flank whilst projecting the tongue, in the absence of an overt stimulus, such as flies. |
| Licking nose | Nose licking was excluded if it occurred after grooming, drinking and eating. |
| Mounting | Includes unsuccessful attempt to mount. |
| Nose wrinkling | Folding of the upper lip and nostrils. |
| Sneeze | Explosive efflux of air. |
| Snorting | Voluntary forced efflux of air through the nose. |
| Standing idle and not approached | The animal was standing idle in the pen on arrival of the observer and did not approach the observing person during the observation. |
| Teeth grinding | Any audible grinding of teeth. |
| Tongue playing or rolling | Stereotypical behaviour. |
| Unusual asymmetrical ear movement | Ears frequently moving independently and asymmetrically in the absence of an obvious continuous auditory stimulus or rapid persistent ear movements (ear flapping) in the absence of an obvious stimulus, such as flies. |
| Vocalisation | Any vocalisation, including grunting and groaning. |
| Yawning | Any yawning regardless of stimulus. |
| ***Sensation*** | |
| Abnormal response to hand approach or clipboard test | Head tossing, nose licking or wrinkling, snorting, startle, backing off to waving a hand (if the animal approaches) or a clipboard (at the end of the observation if the animal does not approach) towards the animal. |
| Head rubbing | Rubbing of the head upon any object within the pen or upon another animal. |
| Hyperaesthesia | Any startle response, either spontaneous or elicited by auditory or visual stimuli. |
| Scratching | Scratching parts of the body with the limbs. |
| Startle at movement including hand approach or clipboard test | Like startle at movements but startle responses to the hand approach or clipboard test are included. |
| Startles at noise | Startle or flinch to auditory stimuli. |
| Startles at movement | Startle of flinch to sudden movement (e.g. arrival of observing person, movement of other animal). |
| ***Movement*** |  |
| Muscle fasciculation | Superficial (skin) muscle contraction which does not cause movement of a body part, in the absence of an obvious stimulus, such as flies. |
| Tremors | Repeated, rhythmical contractions of one or a group of muscles, causing movement but not displacement of the limbs or body. |
